# Validity and reliability of the Malay language Perception Towards Smoking Questionnaire (BM-PTSQ) among secondary school adolescents: Further validation using Confirmatory Factor Analysis

**DOI:** 10.18332/tid/176164

**Published:** 2024-01-24

**Authors:** Kuang Hock Lim, Yoon Ling Cheong, Hui Li Lim, Kee Chee Cheong, Mohd Hazilas Mat Hashim, Ali Aman Marine, Yong Kang Cheah, Jia Hui Lim, Sumarni Mohd Ghazali

**Affiliations:** 1 Institute for Medical Research, National Institute of Health, Ministry of Health Malaysia, Kuala Lumpur, Malaysia; 2 Clinical Research Centre, Hospital Sultan Ismail, Ministry of Health Malaysia, Johor Bahru, Malaysia; 3 Department of Biostatistics, and Data Repository, National Institutes of Health, Shah Alam, Malaysia; 4 School of Economics, Finance and Banking, College of Business, Universiti Utara Malaysia, Sintok, Malaysia; 5 Pharmacy Department, Putrajaya Hospital, Putrajaya, Malaysia

**Keywords:** validation, reliability, Malay version BM-PTSQ, secondary school-going adolescents

## Abstract

**INTRODUCTION:**

Perception is an essential factor influencing smoking among adolescents. Thus, a valid tool for measuring perception is a requisite in smoking studies. This study further establishes the validity and reliability of a Malay language version of the Perception Towards Smoking Questionnaire (BM-PTSQ) for assessing the perception of smoking among secondary school-going adolescents in Malaysia.

**METHODS:**

We administered the BM-PTSQ to 669 secondary school students selected through multistage sampling; 60% of respondents were male (n=398), and 69.9% (n=463) were from rural areas. Respondents were aged 13–16 years, 36.4% (n=241) were 13 years, 40.0% (n=265) were 14 years, and 23.6% (n=156) were 16 years old. We used parallel and exploratory factor analysis (EFA) to determine the domains of the questionnaire. In addition, we also employed EFA, confirmatory factor analyses (CFA), and Cronbach's alpha to evaluate the construct validity and reliability of the BM-PTSQ.

**RESULTS:**

EFA and parallel analysis identified two domains in the BM-PTSQ that accounted for 62.9% of the observed variance, and CFA confirmed the two-domain structure. The two domains' internal consistency scores ranged from 0.702 to 0.80, which suggested adequate reliability.

**CONCLUSIONS:**

The BM-PTSQ has acceptable psychometric validity and is appropriate for assessing smoking perception and intention among Malaysian secondary school-aged youth. Researchers should further evaluate this tool's applicability in a more sociodemographically diverse population.

## INTRODUCTION

Smoking is a behavior that is learned and initiated during adolescence^1,2^, and those who do not begin smoking during their adolescence will most likely not smoke in adulthood. The International Childhood Cardiovascular Cohort (i3C) Consortium, which included seven international cohorts in the US, Australia and Finland recruited in childhood and followed into adulthood, reported a 72.6–88.0% prevalence of smoking among twenty-year-olds who were daily smokers when they were adolescents^3^. Smoking in adolescence increases the risk of diseases related to smoking, such as lung cancer and cardiovascular diseases^4,5^. Therefore, reducing the incidence of smoking among teenagers will have a long-term effect of reducing the future burden of diseases related to smoking^6^. The prevalence of current use of tobacco products was 18.5%, and it was more than three times higher in males (28.0%) than in females (8.9%). Almost 1 in 5 (22.5%) adolescents among the indigenous peoples of Sarawak smoked, whilst the Chinese showed the lowest prevalence (5.2%), and the smoking prevalence increased by age (from 10.7 % among those aged 13 years to 19.1% among school-going adolescents aged 17 years) (95% CI: 8.23–9.62). Regarding e-cigarettes or vaping, males have almost four times higher prevalence compared to females [23.5% (95% CI: 21.61–25.55) vs 6.2% (95% CI: 5.65–6.81)]^7^. However, the prevalence of current use of any tobacco product among adolescents in Malaysia is still high compared to the youth in Indonesia (13.6%), Thailand (14.4%), Philippines (15.8%), and Brunei (17.8%). The use of tobacco products among adolescents in Malaysia in 2022 was 18.5%, slightly lower than 20.9% in 2017^7-11^. Various studies have reported on interpersonal and intrapersonal factors that influence teenagers to start smoking^1,12^. Among the intrapersonal factors are knowledge and perception related to smoking. Studies have shown that there is a positive relationship between perception and smoking initiation^12,13^. These findings align with human behavioral theories such as the Health Belief Model (HBM)^14^ and Protection Motivation Theory (PMT)^15^. Based on this theory, if people perceive the severity of the effects of behavior on health as outweighing the benefits and have no control over it, the likelihood of their practicing the behavior will be low. Therefore, assessment of perception towards smoking, especially among adolescents, is essential to determining the approach to be taken to prevent smoking initiation and thereby reduce the prevalence of health problems related to smoking, especially as smoking-related diseases are the leading causes of mortality and morbidity and among the most significant public health problems in the Malaysian population for the past few decades^16,17^.

Lim et al.^18^ investigated the perception of smoking among teenagers in Malaysia using a 10-item Malay language Perception Towards Smoking Questionnaire (BM-PTSQ) adapted from Grace et al.^19^ in which each item was measured on a 5-point Likert scale (strongly disagree, disagree, agree, and strongly agree). Explanatory Factor Analysis (EFA) of the BM-PTSQ yielded a two-domain solution with satisfactory internal consistency reliability. The variance explained by the two domains was 59.87%. Domain 1 consisted of seven items with a variance explained of 38.25% and Cronbach’s alpha for internal consistency of 0.861, and as for the items in domain 2, the variance explained was 21.62 % and internal consistency alpha was 0.661, all of which indicated sufficient internal consistency. A moderate correlation was observed between domains 1 and 2 (r=0.50). High scores in each domain suggested that the respondents had negative perceptions toward cigarette smoking.

EFA analysis is data-driven and used when the underlying component structure of the instrument is unknown^20^. Even if the best option has been found from EFA that considers one or more qualitative or quantitative factor(s), the resulting instrument is, at best, a potential measurement tool, and further and more thorough examination of the instrument is still required. Hence, in this study, we performed quantitative content validation and CFA analysis of the BM-PTSQ to confirm the findings of Lim et al.^18^ from EFA and to affirm this instrument’s validity and reliability among Malaysian secondary school adolescents.

## METHODS

The data for this study were from a pilot test for the Malaysian Adolescent Health Risk Behavior study (MyAHRB). MyAHRB was a survey among upper secondary school students in Peninsular Malaysia. The pilot study was conducted to verify the construct validity and reliability of several psychometric instruments before their eventual deployment in the survey, the full methodology of which has been described in detail by Lim et al.^21^. To summarize, the pilot test involved 669 secondary school students in the Kota Tinggi district. The pilot questionnaire contained items about sociodemographic variables, the practice of health risk behaviors (smoking, alcohol consumption), the Rosenberg self-esteem scale (RSES), which is a well-known instrument used to measure self-esteem^22^, the Decisional Balance Inventory (DBI), which is a struggle model, a fundamental process of making a decision connected with specific health behaviours^23^, and the Malay-translated version of the Perception Towards Smoking questionnaire (BM-PTSQ). We chose the items in the questionnaire from several sources, i.e. the US Youth Risk Behavior Survey (YRBS), the Global School Health Survey (GSHS) and other published literature on adolescent health behavior.

The BM-PTSQ scale in this study was adopted from Lim et al.^18^. The sample size for the pilot test was the estimated sample size required to compute RMSEA for validation of the DBI. Two-stage, proportionate-to-size sampling was used to recruit a sample of secondary school students in Kota Tinggi district, Johor. The first stage involved systematic random sampling of secondary schools in the district, and then we randomly chose the two classrooms from the schools chosen in the first stage. We invited all the students in the chosen classes to participate in the research.

### Construct validity

**Figure 1:**
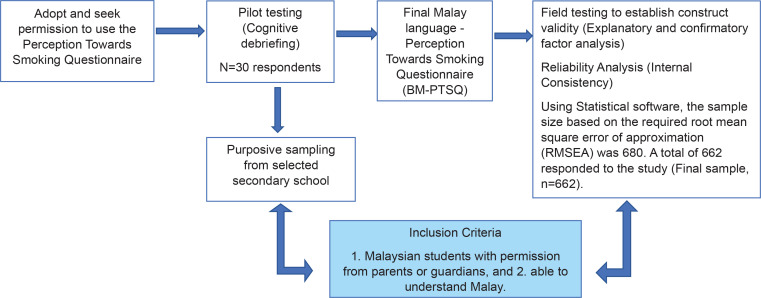
Schematic diagram of the BM-PSTQ validation methodology among secondary school students in Peninsular Malaysia (N=662)

We carried out the validation procedure using the methodology recommended by Wild et al.^24^. This involved assessing construct validity, dependability, and face and content validity (Figure 1). We conducted a cognitive debriefing of the instrument on 30 school-aged teenagers to assess their comprehension, and the level of ambiguity of each item. Feedback from the cognitive debriefing session indicated respondents had no difficulties understanding the items except for one item which appeared ambiguous, i.e. ‘Addiction to smoking is not as strong as addiction to drugs’, and the wording of this item was thus changed to ‘Although addiction to smoking is not as strong as addiction to drugs, it is still harmful’. The amended questionnaire was then sent to six content experts consisting of public health specialists, health education officers, and academics involved in smoking research. The experts evaluated the items in terms of their consistency, relevance, representativeness, and clarity and gave each item a relevance rating ranging from 1 to 4 (where 1=irrelevant, and 4=extremely relevant, very simple, and very clear). After gathering feedback from the experts, the item ‘Most cancers are not preventable and are beyond my control’ was changed to ‘Most types of cancer are preventable, and their occurrence is within our control’. Cognitive debriefing was conducted again with the revised questionnaire, from which positive feedback was obtained (no ambiguity or difficulty with any item in the instrument was reported). The expert review process was repeated on the final version of the questionnaire, and the final ratings were collected.

The final ratings were used to compute several content validity indices: item-level content validity index (I-CVI), scale-level content validity index based on the average method (S-CVI/Ave) and scale-level content validity index based on the universal agreement method (S-CVI/UA). Before calculating the CVIs, the relevance ratings were first grouped into 1 and 0 (1=rating of 3 or 4; and 0= rating of 1 or 2), and subsequent calculations were based on this binary rating.

I-CVI was calculated for each item and is the proportion of experts who gave a relevance rating of 1 for the item (I-CVI = number of experts who gave rating 1 /total number of experts). The S-CVI/Ave is the average of the I-CVI scores for all items (S-CVI/Ave = sum of I-CVI/total number of items). For each item, the universal agreement (U.A.) is also determined wherein the U.A. score is 1 if all experts gave it a rating of 1, and otherwise 0. For each expert, the proportion of relevance was calculated, which is the number of items given a relevance rating of 1 by the expert divided by the total number of items. Given the U.A., the S-CVI/UA can then be computed, i.e. S-CVI/UA = sum of U.A./total number of items. The cut-off value for acceptable I-CVI based on six experts is ≥0.83^25^. Modified kappa for agreement was calculated based on kappa (κ) = (I-CVI - pc)/(1- pc), in which pc (the probability of a chance occurrence) was computed using the formula: pc = 0.6[N!/A!(N-A)!], where N = number of raters and A = number of raters who agree that the item is relevant or clear. Interpretation of κ values, based on guidelines described in Cicchetti and Sparrow^26^, were interpreted as follows: 0.40–0.59 fair, 0.60–0.74 good, and >0.74 excellent. I-CVI and S-CVI/Ave values ≥0.74 were considered excellent, 0.60–0.74 good, and 0.54–0.59 fair.

### Protocol

We sought active consent from the students invited to participate in the study. In this approach, study participant information sheets containing information about the study were sent to the parents and guardians of the selected students. These sheets stated the purpose, scope, voluntary involvement, and use of the data for research purposes and were accompanied by blank consent forms. Only students who returned valid consent forms (written, signed and dated by their parent/s or guardian/s), were Malaysian, understood Malay language, and did not have dyslexia, were enrolled in the survey. A research team member handed the students self-administered paper and pencil questionnaires. Participants were assured that the information disclosed would be kept private and anonymous and that they could withdraw at any time during the study. Each questionnaire took from 20 to 30 minutes for respondents to complete. The Medical Research Ethics Committee, Ministry of Health Malaysia and the Ministry of Education approved this study protocol.

### Data management and analysis

The data were cleaned to remove errors, such as duplication or inconsistencies, before the analyses were performed. The characteristics of the respondents were described using descriptive statistics. Content validity was determined by computing the Item-level Content Validity Index (I-CVI), Scale-level Content Validity Index (SCVI), and kappa statistics for agreement.

We used exploratory factor analysis to evaluate the construct validity of the revised instrument. The criterion for determining the ideal number of domains in EFA was the minimum number required to achieve an eigenvalue exceeding one and visualize the scree plot. Parallel analysis was performed using FACTOR software^27^. The final number of domains was determined by the intersection of the variances obtained in FACTOR and EFA. Varimax rotation was applied to determine the items in each domain, with a cut-off factor loading of 0.3 for item inclusion. We performed the Kaiser-Meyer-Olkin and Bartlett’s tests of sphericity to determine the adequacy of the data.

The results of the EFA were further analyzed by confirmatory factor analysis (CFA) using SPSS AMOS. Six indices were computed to assess model fit: relative χ^2^ (≤5)^28^, Goodness of Fit Index (GFI), Comparative Fit Index (CFI ≥0.090), Root Mean Square Error of Approximation (RMSEA ≤0.080)^28^, Incremental Fit Index (IFI ≥0.090)^28^, and Tucker Lewis index or Non-Normed Fit Index (NNFI) (TLI ≥0.095)^29^. We used CFA to determine construct and discriminant validity, average variance and construct reliability. Examining the overall connection and the effects of eliminating each factor allowed us to determine the instrument’s reliability. Finally, we conducted reliability analyses for each domain in SPSS with the 95% significance threshold used in all statistical analyses. All statistics tests conducted were two-tailed.

## RESULTS

The ten items of the BM-PTSQ are given in Table 1. The results of the final iteration of the BM-PTSQ content validity analysis are shown in Table 2. Five content experts categorized items 1, 4, 6 and 8 with relevance scores of 3 or 4, whereas for items 2, 3, 5 and 7, all six experts gave scores of 3 or 4 (universal agreement). Only half of the content experts evaluated two items (9 and 10) with scores of 3 or 4. These two items were ‘Programs to prevent smoking among teenagers are important’ and ‘My lifestyle and health behaviors, such as eating and smoking habits, will determine whether I get sick or not’. Therefore, these items were excluded from further analysis.

**Table 1:** Final version of the BM-PTSQ tested among secondary school students in Peninsular Malaysia (N=662)

*No.*	*Item*
1	Amalan merokok akan menjejaskan kesihatan saya (Cigarette smoking is harmful to my health)
2	Sekiranya saya merokok, saya akan menghadapi gejala kesukaran bernafas (If I smoke, I will develop breathing problems)
3	Sekiranya saya merokok, saya akan menghadapi gejala penyakit selsema dan jangkitan saluran pernafasan (If I smoke, I can easily get colds or upper respiratory infections)
4	Sekiranya saya merokok, saya mungkin akan menghadapi kanser paru-paru (If I smoke, I may develop lung cancer)
5	Hasil tembakau, sama ada dikunyah atau dihisap akan menyebabkan kanser (Tobacco, whether chewed or smoked, can cause cancer)
6	Meskipun kesan ketagihan hasil tembakau (rokok) tidak sekuat dadah tetapi ianya akan mendatangkan hazard (Although the addictive effect of tobacco (cigarettes) is not as strong as drugs, it will bring hazards)
7	Kebanyakan jenis kanser boleh dicegah dan kejadiannya adalah dalam kawalan kita (Most types of cancer are preventable, and their occurrence is within our control)
8	Wanita mengandung yang merokok akan menjejaskan kesihatan janinnya (If a woman smokes during pregnancy, it will harm the health of her baby)
9	Program mencegah amalan merokok dalam kalangan remaja adalah penting (Programs to prevent smoking among teenagers are important)
10	Sekiranya saya merokok dan tidak bersenam, kesihatan saya akan terjejas di masa depan (My lifestyle and heath behaviors, such as eating and smoking habits, will determine whether I get sick or not)

**Table 2:** BM-PTSQ items relevance ratings by six content experts, validity indices and modified kappa for agreement among secondary school students in Peninsular Malaysia (N=662)

*Items*	*Relevance rating*		*I-CVIs*	*Modified kappa*	*Interpretation*
	*3 or 4*	*1 or 2*			
1	5	1	0.83	0.82	Acceptable
2	6	0	1.00	1	Acceptable
3	6	0	1.00	1	Acceptable
4	5	1	0.83	0.82	Acceptable
5	6	0	1.00	1	Acceptable
6	5	1	0.83	0.82	Acceptable
7	6	0	1.00	1	Acceptable
8	5	1	0.83	0.82	Acceptable
9	3	3	0.50	0.45	Not acceptable
10	3	3	0.50	0.45	Not acceptable

I-CVI: item level content validity index. S-CVI/Ave = 0.915 (S-CVI/Ave = Sum of the I-CVIs/total number of items). S-CVI/UA = 0.50 (S-CVI/UA = Sum of items with I-CVI equal to 1 /total number of items).

The CVI for items 1–8 ranged 0.83–1.0, whereas the modified kappa ranged 0.816–1.00. The average of I-CVI scores across all items (S-CVI/Ave) was 0.915, whilst the average of universal agreement (U.A.) scores across all items (S-CVI/UA) was 0.50, lower by 0.415 compared to S-CVI/Ave.

### Construct validity

A total of 662 students participated in the pilot study, with a high response rate of 98.2%. Nearly 60% (389) of the 662 respondents were male, and over 70% (463) attended secondary schools in rural areas. Most respondents were Malay (86.3%, n=571), and the remaining 10.6% (70) comprised Chinese, Indians, and other ethnic groups. One-third of the respondents were current smokers (smoked at least once in the last 30 days) (Table 3). Parallel analysis and EFA provided a two-domain solution. EFA analysis revealed that the BM-PSTQ consisted of two domains, with an explained variance of 62.9%, Kaiser-Meyer-Olkin measure of sampling adequacy=0.823 and significant Bartlett’s test of sphericity (χ^2^=1493.95, df=28, p<0.001). Domain 1 consisted of five items, accounting for 36.1% of the variance, while the second domain (three items) represented 26.8%. Considering the nature of the items in each domain, we determined that the first domain was mainly concerning the ‘Perception of smoking toward own and others’ health’, whereas the second domain was about the ‘General perception of smoking hazard’ (Table 4).

**Table 3:** Sociodemographic characteristics of study respondents, secondary school students in Peninsular Malaysia (N=662)

*Characteristics*	*n*	*%*
**Gender**		
Male	398	60.1
Female	264	39.9
**Age** (years)		
13	241	36.4
14	265	40.0
16	156	23.6
**Schooling locality**		
Urban	199	30.1
Rural	463	69.9
**Smoking status**		
Current smoker	157	24.6
Non-smoker	482	75.4

**Table 4:** Factor loadings of the BM-PTSQ after varimax rotation among secondary school students in Peninsular Malaysia (N=662)

*No.*	*Item*	*Total variance explained (62.9%)*	
		*Domain 1 (36.1%)*	*Domain 2 (26.8%)*
1	Amalan merokok akan menjejaskan kesihatan saya (Cigarette smoking is harmful to my health)	0.72	
2	Sekiranya saya merokok, saya akan menghadapi gejala kesukaran bernafas (If I smoke, I will develop breathing problems)	0.84	
3	Sekiranya saya merokok, saya akan mudah mendapat gejala penyakit selsema dan jangkitan saluran pernafasan (If I smoke, I will quickly get colds or upper respiratory infections)	0.74	
4	Sekiranya saya merokok, saya mungkin akan menghadapi kanser paru-paru (I might develop lung cancer)	0.77	
5	Wanita mengandung yang merokok akan menjejaskan kesihatan janinnya (If a woman smokes during pregnancy, it will harm the health of her baby)	0.65	
6	Hasil tembakau, sama ada dikunyah atau di hisap akan menyebabkan kanser (Tobacco, whether chewed or smoked, can cause cancer)		0.81
7	Meskipun kesan ketagihan hasil tembakau (rokok) tidak sekuat dadah tetapi ianya masih mendatangkan mudarat (Although the addictive effect of tobacco (cigarettes) is not as strong as drugs, it is still hazardous)		0.73
8	Kebanyakan jenis kanser boleh dicegah dan kejadiannya adalah dalam kawalan kita (Most types of cancer are preventable, and their occurrence is within our control)		0.73

Kaiser-Meyer-Olkin measure of sampling adequacy = 0.823 indicates the cohesion of items in the instrument and suitable for exploratory factor analysis. Bartlett’s test of sphericity χ^2^=1493.95, df=28, p<0.001, indicate that correlations between variables are significant enough and items in the correlation matrix are sufficiently interrelated for factor analysis.

**Figure 2:**
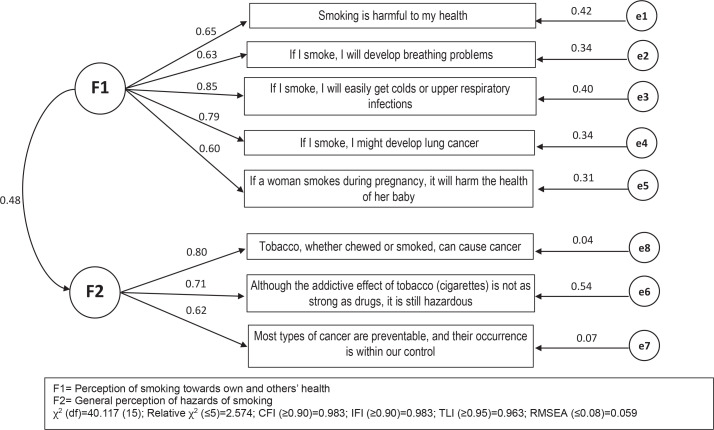
The Malay language Perception Towards Smoking Questionnaire (BM-PTSQ) structural model among secondary school students in Peninsular Malaysia (N=662)

Figure 2 shows the path diagram of the BM-PTSQ CFA model. In the diagram, F1 and F2 circles represent the two domains, the rectangles represent the observed items, single-headed arrows represent the effect of one variable on another variable and the figures alongside the arrows their corresponding correlation coefficients, and the small circles represent unobserved measurement errors for each item. The double-headed arrow represents the covariance or correlation between the two domains. Correlation coefficients between the items and the latent construct ‘Hazard of smoking to an individual and others’ ranged 0.60–0.85. The item ‘If I smoke, I will easily get colds or upper respiratory infections’ had the highest correlation coefficient of 0.85. We observed comparable coefficients in the second domain, all of which exceeded 0.50 (range 0.60–0.80). The item ‘Tobacco, whether chewed or smoked, can cause cancer’ had a correlation coefficient of 0.80, which was the highest in domain 2. Model fit indices showed the CFA model had a reasonable fit. The relative χ^2^=2.674, which is lower than the threshold value (cut-off <5.00), while CFI=0.983 (cut-off ≥0.90), IFI=0.983 (cut-off ≥0.90), TLI=0.963 (cut-off ≥0.95), and the RMSEA=0.059 (cut-off ≤0.08)^28,29^.

## DISCUSSION

This study aimed to validate the BM-PSTQ further among high school students in Kota Tinggi district. After validation, we retained only eight items (Table 5) and two items were removed based on expert opinion, i.e. ‘Preventing teens from cigarette smoking is essential’ and ‘My lifestyle and health behaviors, such as eating and smoking habits, will determine whether I get sick or not’. These two items obtained relevance rating scores of either 3 or 4 from three of the six content experts (50%), thus not meeting the 83% cut-off^21^, which in this case is a minimum of 5 out of 6 experts. One of the comments, given by the expert panel for dropping these items, was that they were not significantly related to the perception of smoking.

**Table 5:** 8-item BM-PTSQ item-total correlation, internal consistency and variance extracted among school-going adolescents in Kota Tinggi, Johor, Malaysia (N=662)

*Domain*	*Item*	*Item-total correlation*	*Internal consistency*	*Average variance extracted*
**Perception of smoking toward own and others’ health**	Amalan merokok akan menjejaskan kesihatan saya (Cigarette smoking is harmful to my health)	0.53	0.80	0.51
	Sekiranya saya merokok, saya akan menghadapi gejala kesukaran bernafas (If I smoke, I will develop breathing problems)	0.64		
	Sekiranya saya merokok, saya akan menghadapi gejala selsema dan jangkitan saluran pernafasan (If I smoke, I will easily get colds or upper respiratory infections)	0.65		
	Sekiranya saya merokok, saya mungkin akan menghadapi kanser paru-paru (If I smoke, I might develop lung cancer)	0.68		
	Wanita mengandung yang merokok akan menjejaskan kesihatan janinnya (If a woman smokes during pregnancy, it will harm the health of her baby)	0.47		
**General perception of hazards of smoking**	Hasil tembakau, sama ada dikunyah atau dihisap akan menyebabkan kanser (Tobacco, whether chewed or smoked, can cause cancer)	0.51	0.70	0.51
	Meskipun kesan ketagihan hasil tembakau (rokok) tidak sekuat dadah tetapi ia masih mendatangkan mudarat (Although the addictive effect of tobacco (cigarettes) is not as strong as drugs, it still hazardous)	0.54		
	Kebanyakan jenis kanser boleh dicegah dan kejadiannya adalah dalam kawalan kita (Most types of cancer are preventable and their occurrence is within our control)	0.51		

All eight items reached the minimum required CVI level of 0.83, and the modified kappa coefficient exceeded 0.80. Construct validity via EFA and parallel analysis suggested a 2-domain solution, with the first domain consisting of 5 items that explained 36.1% of the variance. In contrast, the second domain consisted of three items, which explained 27.9% of the variance. This finding is consistent with the results reported by Lim et al.^18^ in a study conducted in the same locality. The explained variance in the initial questionnaire exceeded the required minimum of 50%^30^, and the CFA findings supported the results of parallel analysis and EFA. The subscales’ item-to-subtotal correlation coefficients in domain 1 were 0.60–0.85, and in domain 2, 0.63–0.80, i.e. all were above 0.5, which indicates a fair degree of reliability. In addition, a high correlation can also be observed between domains 1 and 2, which shows that these two domains are related to each other.

In this study, the items in domain 1 and domain 2 are almost identical to those in Lim et al.^18^, and the amount of explained variance in both studies is almost the same. However, the present study’s internal consistency for domain 1 was lower than that of Lim et al.^18^ (Cronbach alpha value of 0.861 versus 0.800). The lower Cronbach alpha might be because the number of items in the Lim et al.^18^ study is seven compared to five items in a similar domain; in addition, the lower Cronbach alpha in our study may also be due to the total correlation for the item ‘If a woman smokes during pregnancy, it will harm the health of her baby’, which is rather low compared to other items in domain 1 (0.626–0.677 compared to only 0.475 for this item). This item may reflect the fetal risk of maternal smoking. In contrast, the other four items were the perception of risk to oneself. However, this item has an acceptable level of correlation (0.30). Our findings demonstrated that the modified BM-PTSQ is able to distinguish between two categories of perception (perception of the hazard of smoking to oneself and general perception of the effects of smoking), CFA of the BM-PTSQ revealed there is a positive link between the two, with a moderate correlation. The BM-PTSQ can also gauge various perceptions of the drawbacks of smoking, including worries about its negative health impacts. These findings indicate that researchers and public health advocates may use the BM-PTSQ instrument to measure the perception of adolescents towards smoking, which, in turn, may be useful in formulating specific measures, policies and interventions to reduce smoking initiation and increase the smoking cessation rate among adolescents.

### Limitations

A limitation of this study is that it was conducted among secondary school students in the Kota Tinggi District. Hence, we cannot infer that the study findings apply to Malaysia’s general population of adolescents. Secondly, adding more culturally relevant items to the questionnaire may increase its validity, as Malaysian youth culture may differ from the population for which the original PTSQ was initially developed. In addition, concurrent and discriminant analyses were not carried out in the study. Further studies should be conducted to validate this instrument using other methods, such as item response theory or Rasch analysis.

## CONCLUSIONS

Overall, the study findings confirm the validity and reliability of the BM-PSTQ, albeit with minor modifications, indicating it is a reliable and valid instrument for assessing Malaysian adolescents’ perception of smoking. Additional testing in a more sociodemographically and geographically diverse sample of adolescents is necessary to ensure the BM-PSTQ is a valid measure of smoking perception in the general Malaysian adolescent population.

## Data Availability

The data supporting this research are available from the authors on reasonable request.
